# Detection of Trace Amounts of Water in Organic Solvents by DNA-Based Nanomechanical Sensors

**DOI:** 10.3390/bios12121103

**Published:** 2022-12-01

**Authors:** Tomohiro Murata, Kosuke Minami, Tomohiko Yamazaki, Genki Yoshikawa, Katsuhiko Ariga

**Affiliations:** 1 Graduate School of Frontier Sciences, The University of Tokyo, 5-1-5 Kashiwanoha, Kashiwa 277-8561, Japan; 2 International Center for Materials Nanoarchitectonics (MANA), National Institute for Materials Science (NIMS), 1-1 Namiki, Tsukuba 305-0044, Japan; 3 Center for Functional Sensor & Actuator (CFSN), Research Center for Functional Materials (RCFM), National Institute for Materials Science (NIMS), 1-1 Namiki, Tsukuba 305-0044, Japan; 4 Research Center for Functional Materials (RCFM), National Institute for Materials Science (NIMS), 1-2-1 Sengen, Tsukuba 305-0047, Japan; 5 Division of Life Science, Hokkaido University, Kita 10, Nishi 8, Kita-ku, Sapporo 060-0808, Japan; 6 Materials Science and Engineering, Graduate School of Pure and Applied Science, University of Tsukuba, 1-1-1 Tennodai, Tsukuba 305-8571, Japan

**Keywords:** DNA, nanomechanical sensors, membrane-type surface stress sensor (MSS), water detection, trace amounts of water in organic solvent

## Abstract

The detection of trace amounts of water in organic solvents is of great importance in the field of chemistry and in the industry. Karl Fischer titration is known as a classic method and is widely used for detecting trace amounts of water; however, it has some limitations in terms of rapid and direct detection because of its time-consuming sample preparation and specific equipment requirements. Here, we found that a DNA-based nanomechanical sensor exhibits high sensitivity and selectivity to water vapor, leading to the detection and quantification of trace amounts of water in organic solvents as low as 12 ppm in THF, with a ppb level of LoD through their vapors. Since the present method is simple and rapid, it can be an alternative technique to the conventional Karl Fischer titration.

## 1. Introduction

DNA is one of the most interesting biopolymers in science. Besides its central function as the storage and carrier of genetic information, its unique nature offers great potential in many fields. One of the most important properties of DNA is its hybridization capability, such as the ability to form double helices and guanine quadruplexes. DNA nanotechnology has been extensively studied [1,2,3], and versatile potential applications including therapeutics [4,5] and diagnosis [6,7,8] have been reported. In particular, DNA hybridization capability has been exploited in the field of biosensors to detect nucleic acids with high selectivity [9,10,11,12]. Another important property of DNA is its hydration behavior [13]. The hydration/dehydration behavior is known to affect the hybridization process and the derived structures [14,15,16,17] as well as the material properties, such as the mechanical characteristics [15,18,19].

Focusing on the changes in mechanical properties associated with DNA hydration, nanomechanical sensors have attracted attention as potential sensing platforms to detect the mechanical deformation of receptor layers caused by the sorption of target molecules and hence have been applied to measuring DNA hydration [17,18,20], mechanical properties [18,19] and hybridization [18,21]. The hybridization behavior of DNA has led to the development of DNA-based nanomechanical sensors for various applications [22,23,24,25,26]. Although there is a limited number of papers using DNA as a receptor material for nanomechanical sensors to detect water vapor, albeit for different purposes (i.e., the detection of hydration) [17,20], DNA-based nanomechanical sensors have mostly been applied in aqueous environment because of their potential applications [24,25]. 

Moreover, in hydration, a limited number of water molecules hydrate the phosphate groups in the DNA backbone [13]. Since this hydration can be detected by nanomechanical sensors [17,18,20], it has great potential as a sensor to detect trace amounts of water, especially in organic solvents, which is of great importance in the field of chemistry and in the industry. Although there are several methods to detect trace amounts of water in organic solvents, such as conventional coulometric and volumetric analyses, known as Karl Fischer titration [27,28,29], as well as colorimetric approaches [30,31] and others [32,33], to the best of our knowledge, there have been few reports on detecting trace amounts of water in organic solvents through their vapors.

In this work, we found that a DNA-based nanomechanical sensor exhibited high selectivity to water vapor. We used one of the nanomechanical sensors operating in the so-called static mode, a Membrane-type Surface stress Sensor (MSS) [26,34], and deposited natural DNA obtained from salmon testes onto the MSS. Compared to nanomechanical sensors in dynamic mode operation [25] as well as to other types of gas and chemical sensors [35], nanomechanical sensors in static mode operation detect mechanical stress/strain [26] and hence transduce the mechanical deformation induced by the swelling of a DNA layer associated with DNA hydration, resulting in high sensitivity to water. Owing to the high sensitivity and selectivity of the examined sensor to water vapor, we also demonstrate in this work that trace amounts of water in organic solvents can be detected. This study not only presents the possibility of using DNA as a water-sensitive receptor material but also provides a promising sensing platform for detecting trace amounts of water in organic solvents as an alternative to the conventional Karl Fischer titration method [27,28,29].

## 2. Materials and Methods

### 2.1. Materials

DNA from salmon testes was purchased from Tokyo Chemical Industry CO., LTD. Ultrapure water for inkjet spotting and sensing measurements was prepared by Millipore Milli-Q. Acetone, methanol, ethanol, acetonitrile, *n*-hexane, benzene, toluene, tetrahydrofuran (THF), and pyridine for sensing measurements were purchased from FUJIFILM Wako Pure Chemical Corporation. All solvents were dehydrated (H_2_O < 0.001–0.005%). Molecular sieves 3A were purchased from FUJIFILM Wako Pure Chemical Corporation. Unless otherwise noted, the materials were used as purchased.

### 2.2. Fabrication of DNA-Coated MSS

**Figure 1:**
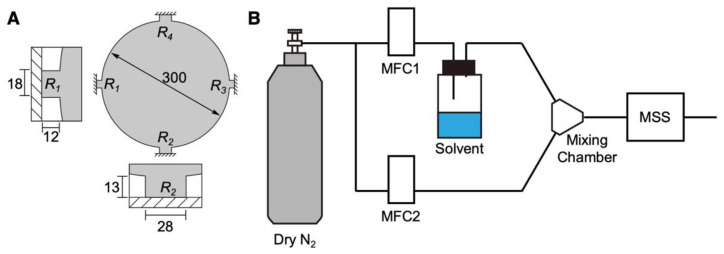
Schematic illustrations of the MSS and its sensing system. (**A**) Configuration of the MSS. Sensing beams with embedded piezoresistors are magnified in the insets. Numbers indicate the dimensions in µm. (**B**) Schematic illustration of the measurement system.

The construction of the MSS chips and their working principle have been previously reported [34,36]. Briefly, the MSS consists of a silicon-based membrane suspended by four sensing beams, composing a full Wheatstone bridge (Figure 1A). In each sensing beam, piezoresistors were embedded by boron doping. The membrane is coated with a receptor material (in the present study, DNA). When the receptor layer deforms upon the sorption of the target analytes, the receptor layer generates surface stress on the membrane [26]. The surface stress is transduced to the four sensing beams as amplified uniaxial stress, resulting in changes in the electrical resistance of the piezoresistors embedded in the beams. The signal output of the MSS (*V*_out_) is provided by the total output resistance change obtained from the Wheatstone bridge circuit; it can be expressed as

(1) $$V_{\mathrm{out}}=\frac{V_{B}}{4}\left(\frac{\Delta R_{1}}{R_{1}}-\frac{\Delta R_{2}}{R_{2}}+\frac{\Delta R_{3}}{R_{3}}-\frac{\Delta R_{4}}{R_{4}}\right)$$

where *V_B_* is the bridge voltage applied on the Wheatstone bridge circuit, and Δ*R/R_i_* (*i* = 1–4) is the relative resistance change in each sensing beam. 

The MSS chips used in the present study were purchased from NanoWorld AG, Switzerland. The dimensions of the MSS used in this study are shown in Figure 1A. Before the deposition of DNA, the MSS chips were treated with oxygen plasma using a low-pressure plasma system (Femto, version B, Diener Electronic GmbH + Co. KG., Ebhausen, Germany). The plasma power, pressure, and duration were 30 W, 0.50 mbar, and 2 min, respectively. DNA was deposited directly on the membrane of the MSS by using an inkjet spotter (LaboJet-500SP, MICROJET Corporation, Nagano, Japan) with a nozzle (IJHBS-300, MICROJET Corporation). DNA was dissolved in ultrapure water at a concentration of 0.2 mg mL^−1^, and the resulting solution was deposited onto each channel of the MSS. The discharged volume per shot by the inkjet was 492 ± 15 pL (*n* = 3) at this concentration.

### 2.3. Characterization of the DNA Film

The thickness and surface profile of the DNA films coated on the MSS were measured by using a 3D surface profiler (VK-X3000, KEYENCE Corporation, Osaka, Japan) under the laser confocal mode and a surface stylus profiler (DekTak, Bruker). To confirm the structure of DNA, the circular dichroism (CD) spectra of an aqueous solution and a drop-casted film of DNA were measured using a spectropolarimeter (J-820, JASCO). An aqueous solution of DNA (5 mg mL^−1^) was drop-casted on a quartz substrate. The base-pair lengths of the DNA molecules were confirmed by electrophoresis. Electrophoresis was performed using a polyacrylamide gel (e-PAGEL 10–20%, ATTO) in TG buffer at 21 mA for 65 min (WSE-1150 PageRunAce, ATTO). Ultra-Low-Range DNA Ladder (Thermo Fisher Scientific) was used for the quantification based on base size. The DNA samples were stained for 30 min in TBE buffer with SYBR™ Gold (Thermo Fischer Scientific, Waltham, MA, USA).

### 2.4. Preparation of Dehydrated Organic Solvents and Water-Contaminated Solvents

Dehydrated organic solvents were stored with molecular sieves 3A under nitrogen during the experiment; the molecular sieves were activated before use by heating in an oven at 250 °C overnight and then cooling to room temperature under vacuum for 3 h. To prepare the water-contaminated solvents, an aliquot of water was added to the dehydrated organic solvents. To obtain a series of low concentrations, the resulting water-contaminated solvents were further diluted with the dehydrated organic solvents prepared above.

### 2.5. Vapor Sensing

The vapor sensing system is shown in Figure 1B. The DNA-coated MSS chips were placed in a Teflon-based chamber. The chamber was connected to two gas lines: an inlet and an outlet. The inlet was connected to a gas system, which consisted of two mass flow controllers (MFCs), a mixing chamber, a purging gas line, and a sampling gas line with a vial for a solvent liquid. The gas flow system with the chamber was placed in an incubator, and the temperature was maintained at 25.0 ± 0.5 °C. The headspace vapor of each solvent was introduced by a carrier gas. Dry and pure nitrogen gas was used as a carrier and purging gas. The duration was precisely controlled using the two MFCs. Before measuring the MSS output, pure nitrogen gas was introduced into the MSS chamber for at least 5 min (i.e., MFC-2 at 100 mL min^−1^) to promote the desorption of molecules absorbed in the previous measurement. Subsequently, MFC-1 (injection line) was controlled at 80 mL min^−1^ for 5 min and then switched off (i.e., 0 mL min^−1^) for 5 min. The total flow rate was adjusted to 100 mL min^−1^ by controlling MFC-2 during the sensing experiments. The measurement sequence is shown in Figure S1. The data were obtained with the applied bridge voltage *V*_B_ of –0.5 V and recorded at a rate of 20 Hz.

To investigate the selectivity of DNA to various vapors, each vapor was introduced at the concentration *P_a_*/*P_o_* of 0.8, where *P_a_* and *P_o_* denote the partial vapor pressure and saturated vapor pressure of each solvent, respectively. For the measurement of trace amounts of water in organic solvents, each vapor of organic solvents with 0–4000 ppm water added by weight was injected to the chamber.

### 2.6. Measurement of the Humidity Dependence

The signal changes of the DNA-deposited MSS at several humidity values were examined. Water was set as the solvent (Figure 1), and the value of MFC-1 was changed in the range of 0–90% RH. The total flow rate was adjusted to 100 mL min^−1^ by MFC-2. To estimate the strain of the DNA membrane due to water adsorption, a quartz crystal microbalance (QCM; QCM922A, SEIKO EG&G) measurement of the several humidity conditions was also conducted. An AT-cut quartz crystal resonator (QA-A9M-AU(M), SEIKO EG&G) was used and held into a QCM chamber (QA-CL6, SEIKO EG&G) instead of the MSS chamber, as shown in Figure 1. The same amount of DNA solution used for the MSS was deposited on the resonator by the inkjet method mentioned before.

### 2.7. Numerical Simulation

The signal responses of nanomechanical sensors operating in static mode including MSS are obtained by measuring the mechanical stress/strain induced by the sorption of target molecules in a receptor layer (in this study, DNA). Generally, the volume of absorbed molecules is one of the important factors to generate mechanical strain in a receptor layer. To simulate the effects of the volume-dependent strain of absorbed water on the MSS responses, we performed finite element analysis (FEA) using COMSOL Multiphysics 5.6 with the Structural Mechanics module according to our previous studies [37,38]. The dimensions of the MSS are shown in Figure 1A. The volume of absorbed water molecules was estimated through the QCM measurement. Each structure of the DNA-coated MSS was meshed with 10,000–20,000 elements to ensure numerical accuracy for identifying mechanical deformation upon the applied strain and corresponding resistance changes.

## 3. Results and Discussion

### 3.1. Fabrication of the DNA-Coated MSS

**Figure 2:**
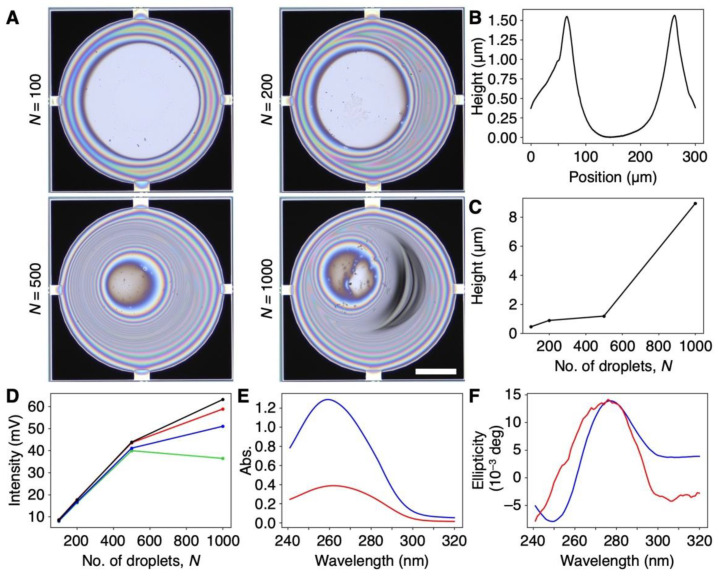
DNA-coated MSS. (**A**) Optical microscope images of the DNA-coated MSS with a different number of inkjet droplets (*N*) ranging from 100 to 1000. Scale bar is 100 µm. (**B**) Height profile of the DNA film on MSS. *N* = 500. See also Figure S2 for all height profiles. (**C**) Plot of maximum thickness of DNA coated on the MSS as a function of a number of inkjet droplets (*N*). (**D**) Sensitivity to water as a function of DNA film thickness. The relative humidity varied as follows: 20%RH (green), 40%RH (blue), 60%RH (red), and 80%RH (black). (**E**,**F**) UV–Vis (**E**) and CD spectra (**F**) of a DNA aqueous solution (60 μg/mL) (blue) and DNA cast on quartz (red). CD spectra of DNA cast on quartz, magnified 10 times.

Since nanomechanical sensors including the MSS can obtain efficient signal responses to sorption-induced mechanical deformation of a bulk receptor material [26,39], we coated DNA on the membrane of an MSS by an inkjet spotter. The coating thickness was varied by changing the number of droplets of inkjet spotting (*N*) in the range from 100 to 1000. Optical laser microscope images are shown in Figure 2A. From the height profiles of the DNA films, the thicknesses of the DNA films were estimated to be approximately 100 nm to 10 µm (Figure 2B,C and Figure S2 in the Supplementary Materials). Although the DNA films on the MSS formed a coffee ring structure, as can be seen in Figure 2B, an MSS is sufficiently robust with respect to the coating quality and inhomogeneity of a receptor layer owing to its symmetric geometry, and the signal deviation is in the range of only 5–6% even with a receptor layer having a coffee ring structure [40]. We used these DNA-coated MSS for further experiments. To verify the thickness-dependent sensitivity of the DNA-coated MSS, we measured the signal responses to water vapor. As shown in Figure 2D, the intensity monotonically increased with a linear correlation up to *N* = 500, whereas the intensity for *N* = 1000 largely deviated, and the intensity at 20% RH was lower than that for *N* = 500. Thus, we used a DNA-coated MSS with *N* = 500 in the following experiments.

We also characterized the DNA used in this work. Since the natural DNA from salmon testes used in this work was used as purchased, the sample contained DNA fragments of various lengths, as shown in Figure S3. According to the CD spectra, the DNA cast on quartz maintained its double-helix structure (Figure 2E,F). During inkjet spotting and the sensing measurements, aqueous solutions of DNA and the DNA-coated MSS were not subjected to any heating processes, suggesting that the DNA maintained its double-helix structure on the MSS.

### 3.2. Selectivity of the DNA-Coated MSS

**Figure 3:**
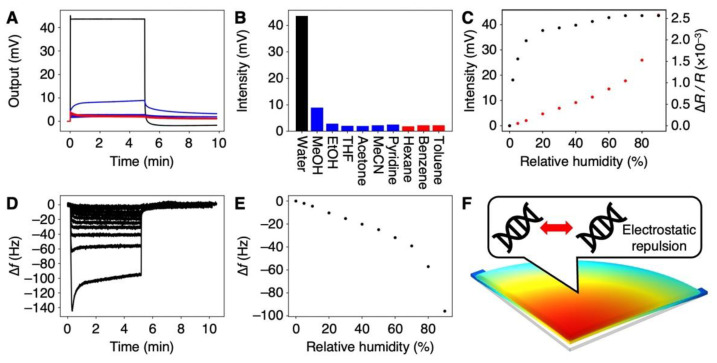
Moisture-selective response of the DNA-coated MSS. (**A**) Signal responses to various vapors at the concentration of *P_a_*/*P_o_* = 80%. Signal responses to all vapors; water (black), water-miscible organic solvents (blue), and water-immiscible organic solvents (red). All responses are shown in Figure S4. (**B**) Signal intensities for each vapor. (**C**) Humidity-dependent responses in the range of 0–80% RH (black). Red plots indicate the FEA results. (**D**) Frequency shifts of DNA-coated QCM in the range of 0–90%RH. (**E**) Frequency shifts of DNA-coated QCM as a function of relative humidity. (**F**) Plausible mechanism of hydration-induced electrostatic repulsion.

To estimate the selectivity of the DNA-coated MSS to water, we measured 10 different vapors, including 6 water-miscible organic solvents (i.e., methanol, ethanol, THF, acetone, acetonitrile, and pyridine), 3 water-immiscible solvents (*n*-hexane, benzene, and toluene), and water. The signal responses of the DNA-coated MSS to each vapor are shown in Figure 3A (see also Figure S4), and the corresponding signal outputs at 5 min after injection (denoted as signal intensity) are summarized in Figure 3B. As clearly seen in Figure 3A,B, the DNA-coated MSS exhibited a significantly high sensitivity to water with Δ*R*/*R*~0.35 (*V*_out_~44 mV; see also Equation (1)), while the sensitivity to other organic solvents was ca. 15–20 times lower, despite their hydrophilicity as well as water-miscibility. Interestingly, the response to benzene, which has a planer structure and is known as a DNA intercalator, exhibited a similar intensity to those measured for other organic solvent vapors. According to the working principle of nanomechanical sensors in static mode operation, the signal output is obtained by the surface stress induced by mechanical deformation [26]. Thus, the intercalation of benzene between the base pairs of DNA seemed to have less effect on mechanical deformation (i.e., expansion of DNA structure), resulting in a low signal response.

We further investigated this remarkable response of the DNA receptor layers to water. The relative humidity (RH) was varied in the range of 0–80%, and the corresponding signal responses were measured (Figure 3C). The signal intensity rapidly increased in the range of 0% to 10% and then it gradually increased. It should be noted that the signal intensity even at 2% RH was 18.1 mV, significantly higher than those for all vapors of organic solvents at *P_a_*/*P_o_* = 80%. Since the theoretical noise of the MSS is ca. 1 µV [36] and the experimentally observed noise was ca. 5 µV, the Limit of Detection (LoD) [41] was estimated as 1.7 × 10^−5^% RH. Compared to other humidity sensors [42], the DNA-based MSS exhibits high sensitivity to water (Table S1 in the supplementary materials). This remarkable selectivity and sensitivity of the DNA-coated MSS to water has a great potential for the detection of trace amounts of water in organic solvents.

To further investigate the mechanism of the selectivity to water vapor, we estimated the hydration-induced strain by using a QCM, followed by a numerical simulation through FEA. The same volume of DNA solution was cast on a QCM. Frequency shifts were measured with varied relative humidity in the range from 0% RH to 90% RH (Figure 3D). The frequency shifts at 5 min after moisture injection are plotted in Figure 3E. As clearly seen in comparison with Figure 3C,E, the trend of the QCM was different from the trend of the MSS. The signal intensity of the MSS rapidly increased up to 10% RH, while the frequency shift of the QCM exhibited linear correlation up to 70% RH, decreasing at the rate of 0.53 Hz per % RH. According to Sauerbrey’s equation [43], we estimated the weight of the absorbed water molecules from the frequency shifts of the QCM. Assuming that the specific volume of absorbed water in the DNA film does not change, the hydration-induced strain can be estimated as 2.5 × 10^−5^ strain per % RH. By using this calculated strain, the trend in the signal output was numerically simulated through FEA (Figure 3C, red). Compared to the FEA results as well as to the QCM trend, the humidity-dependent signal intensity of the DNA-coated MSS was different, especially at the lower relative humidity (i.e., up to 10% RH). In a DNA layer, where water is present, repulsive hydration forces between phosphate groups have been reported [21,44]. These repulsive hydration forces may contribute to the mechanical deformation of the DNA film, resulting in its remarkable sensitivity to water. 

### 3.3. Detection of Trace Amounts of Water in Organic Solvents

Most organic solvents are frequently contaminated with water. Such water, particularly trace amounts of water, has a great influence on chemical reactions [30,45]. Thus, the detection and quantification of trace amounts of water in organic solvents are of crucial importance in chemistry and in the industry. Karl Fischer titration is a commonly used method for determining the water content based on coulometric and volumetric analyses [27,28,29], whereas it also has some drawbacks in terms of rapid and direct detection because of its time-consuming sample preparation and need of specific equipment [46,47]. Although some of the alternative approaches including colorimetry [31], fluorometry [30,31], electrochemistry [32], and nuclear magnetic resonance [33] have been reported, there are still some limitations such as low sensitivity, requirement of expensive equipment, and some insufficiencies in probe materials. In contrast to the conventional Karl Fischer titration as well as to other reported methods, the DNA-based nanomechanical sensors presented in this study can provide an alternative method for detecting and quantifying such trace amounts of water.

**Figure 4:**
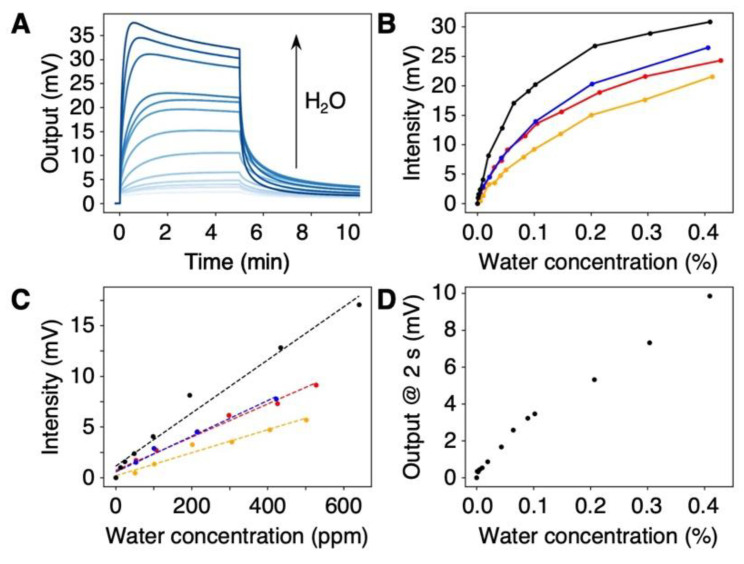
Quantification of trace amounts of water in various organic solvents. (**A**) Concentration-dependent signal responses to water in THF. The signal responses to other solvents are summarized in Figure S5. (**B**,**C**) Plots of signal intensities as a function of water concentration in the ranges of 0–0.4% (**B**) and 0–600 ppm (**C**) with different organic solvents: THF (black); acetone (red); acetonitrile (blue); and pyridine (orange). (**D**) Plot of signal output to THF, 2 s after injection.

To explore the possibility of using the DNA-coated MSS for the quantification of trace amounts of water in organic solvents, we measured the vapors of water-contaminated organic solvents. We prepared the dehydrated organic solvents by dehydration with molecular sieves. Subsequently, an aliquot of water was added to prepare a wide range of water concentrations up to 0.4 *w*/*w*% (i.e., 4000 ppm). Four water-miscible organic solvents were examined: THF, acetone, acetonitrile, and pyridine. The signal responses are depicted in Figure 4A and Figure S5. The signal intensity measured for each organic solvent clearly depended on the concentration of water in the organic solvents in the measured range (Figure 4B), with a linear correlation for the low concentration range (Figure 4C). In the case of THF, the DNA-coated MSS yielded an intensity of ca. 2 mV at a 12 ppm water content. Since the theoretical noise level of the MSS is 1 µV [36] and the experimental noise level was ca. 5 µV, the present system is capable of detecting water at the parts-per-billion (ppb) level in organic solvents (Table S2).

Compared to Karl Fischer titration, the water content could be quantified in a shorter time, e.g., in 30 s after vapor injection (Figure S5C). Furthermore, in the case of THF, the signal output at 2 s after vapor injection allowed us to quantify the water content, as shown in Figure 4D. More importantly, the DNA-coated MSS can be utilized for the quantification of water in pyridine. Since the chemical reaction used in Karl Fischer titration typically requires a base reagent (e.g., pyridine) [27,28,29], such solvents are generally impossible to quantify by Karl Fischer titration. Therefore, DNA-based nanomechanical sensors offer a promising alternative to Karl Fischer titration, providing a simple and rapid method for detecting and quantifying trace water contents in various organic solvents.

### 3.4. Reproducibility of the DNA-Based MSS

In practical applications, the reproducibility of the sensing performance is an important factor. To investigate the stability and reproducibility of the DNA-based MSS, we repeated the sensing experiments to detect water vapor and trace amounts of water in an organic solvent. Water vapor at 80% RH and vapor of 100 ppm water in THF were measured for 50 cycles. As can be seen in Figure S6, the signal intensities in the detection of water vapor and trace amounts of water were 45.97 ± 0.03 mV and 6.81 ± 0.34 mV, respectively. These results clearly indicated that the DNA-based MSS has high stability and reproducibility when detecting the vapors of water as well as trace amounts of water in chemicals, suggesting its practical applicability.

## 4. Conclusions

We demonstrated that a DNA-coated MSS exhibited high sensitivity and selectivity to water, leading to the facile and rapid detection of trace amounts of water in organic solvents. Taking advantage of nanomechanical sensors in the static mode operation, the MSS can efficiently and selectively detect the mechanical deformation of a DNA layer induced by water absorption. This high sensitivity and selectivity enable DNA-coated MSS to detect trace amounts of water in organic solvents as low as 12 ppm (i.e., 0.0012 *w*/*w*%). Although the mechanism of water sensitivity needs further investigation, given the robustness and compactness of the MSS sensing system [26] and its high stability and reproducibility, this study provides a simple and rapid method for the detection and quantification of trace amounts of water in organic solvents as an alternative to the conventional Karl Fischer titration. Furthermore, this method can provide a promising sensing platform for the on-site detection of water, with a large variety of potential applications in various fields including chemistry, food, environment, healthcare, and medicine.

## 5. Patents

T.M., K.M., T.Y., G.Y., and K.A. are inventors, Japanese patent application number 2022-148684, submitted by National Institute for Materials Science (NIMS).

## Data Availability

Not applicable.

## Supplementary Materials

The following supporting information can be downloaded at: https://www.mdpi.com/article/10.3390/bios12121103/s1, Figure S1: Sensing sequence; Figure S2: Surface profile of the DNA film on the MSS; Figure S3: Electrophoresis; Figure S4: Signal responses to organic solvent vapors; Figure S5: Signal responses to water in organic solvents; Figure S6: Reproducibility; Table S1: Limit of Detection to humidity [34,36,42,48,49,50,51,52,53,54,55,56,57,58,59,60,61]; Table S2: Limit of Detection to trace amounts of water in organic solvents.
