# Supplementary material for: Detection of Trace Amounts of Water in Organic Solvents by DNA-Based Nanomechanical Sensors
Source: Biosensors (Basel). 2022 Dec 1;12(12):1103. doi: 10.3390/bios12121103 (PMC9775023; doi:10.3390/bios12121103)
Supplement: Supplementary file 1 [file biosensors-12-01103-s001.zip › biosensors-2033736-supplementary.pdf]

Supplementary materials

Detection of Trace Amounts of Water in Organic Solvents by DNA-Based Nanomechanical Sensors

Tomohiro Murata [1,2], Kosuke Minami [3,*], Tomohiko Yamazaki [4,5], Genki Yoshikawa [3,6] and Katsuhiko Ariga [1,2,*]

[1] Graduate School of Frontier Sciences, The University of Tokyo, 5-1-5 Kashiwanoha, Kashiwa, Chiba 277-8561 Japan; t.murata20s@ams.k.u-tokyo.ac.jp
[2] International Center for Materials Nanoarchitectonics (MANA), National Institute for Materials Science (NIMS), 1-1 Namiki, Tsukuba, Ibaraki 305-0044 Japan
[3] Center for Functional Sensor & Actuator (CFSN), Research Center for Functional Materials (RCFM), National Institute for Materials Science (NIMS), 1-1 Namiki, Tsukuba, Ibaraki 305-0044 Japan; yoshikawa.genki@nims.go.jp
[4] Research Center for Functional Materials (RCFM), National Institute for Materials Science (NIMS), 1-2-1 Sengen, Tsukuba, Ibaraki 305-0047 Japan; yamazaki.tomohiko@nims.go.jp
[5] Division of Life Science, Hokkaido University, Kita 10, Nishi 8, Kita-ku, Sapporo 060-0808, Japan
[6] Materials Science and Engineering, Graduate School of Pure and Applied Science, University of Tsukuba, 1-1-1 Tennodai, Tsukuba, Ibaraki 305-8571 Japan
* Correspondence: minami.kosuke@nims.go.jp (K.M.); ariga.katsuhiko@nims.go.jp (K.A.)

Supplementary Figures

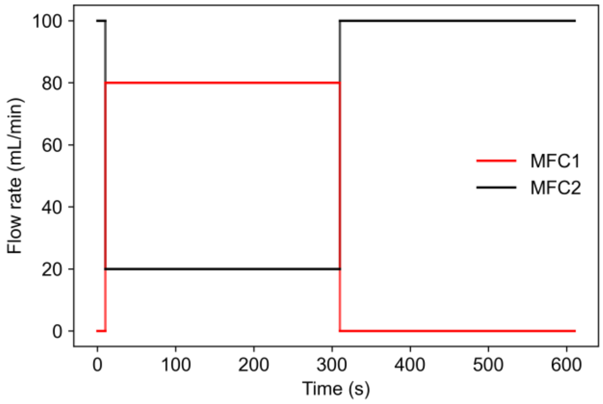

Figure S1. Sensing sequence of mass flow controllers (MFCs).

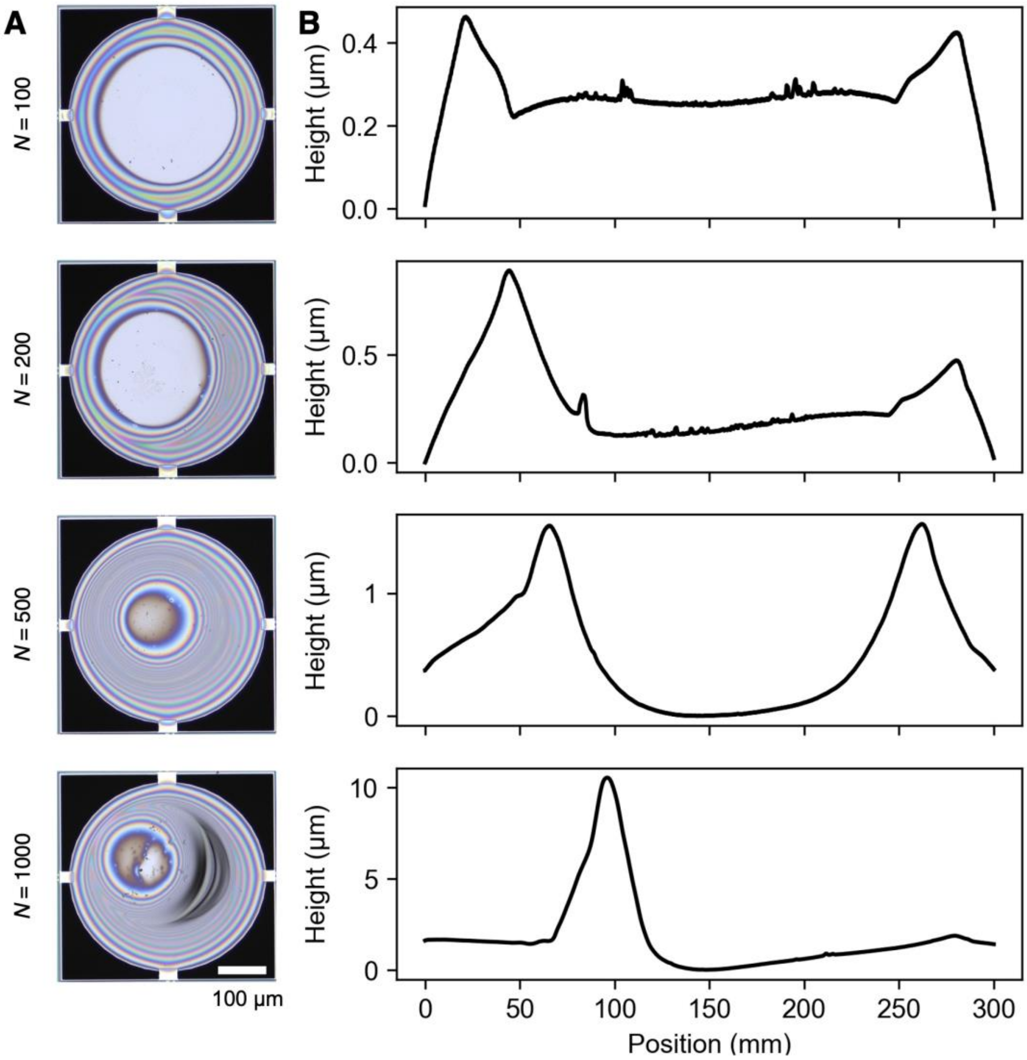

Figure S2. Surface profile of the DNA films coated on MSS.

A, Optical microscope mages of the DNA films with different number of inkjet droplets (N). **B**, Surface profile of the DNA films on MSS measured by surface stylus profiler.

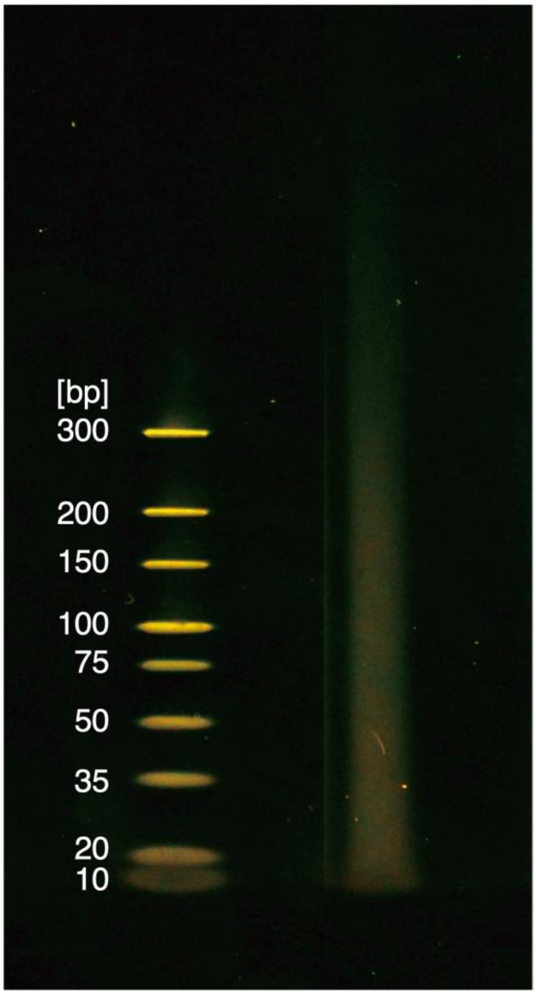

Figure S3. Electrophoresis of calf thymus DNA used in this work.

Polyacrylamide gel electrophoresis (e-PAGEL) in TG buffer. DNA was stained with SYBR Gold.

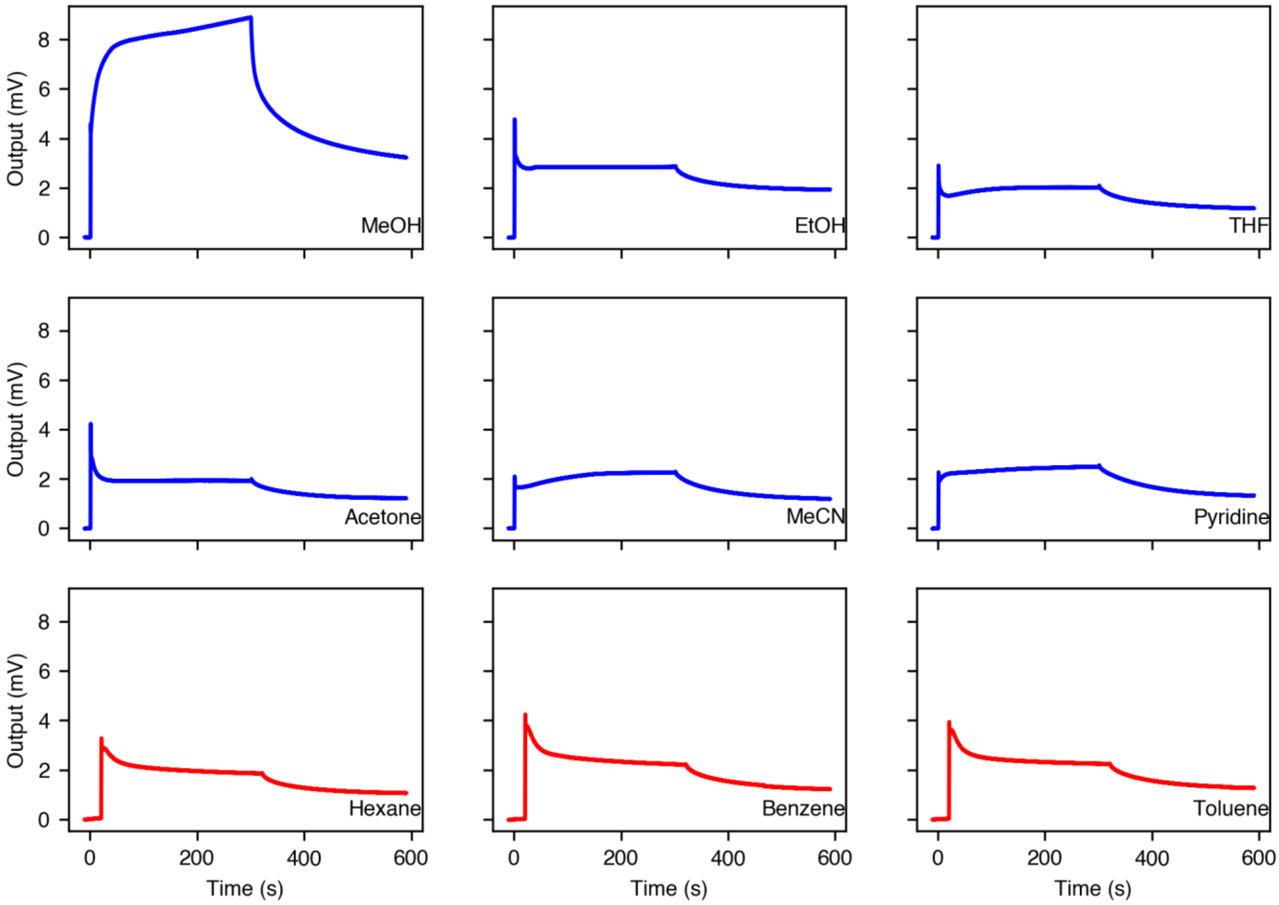

Figure S4. Signal responses to various vapors of organic solvents.

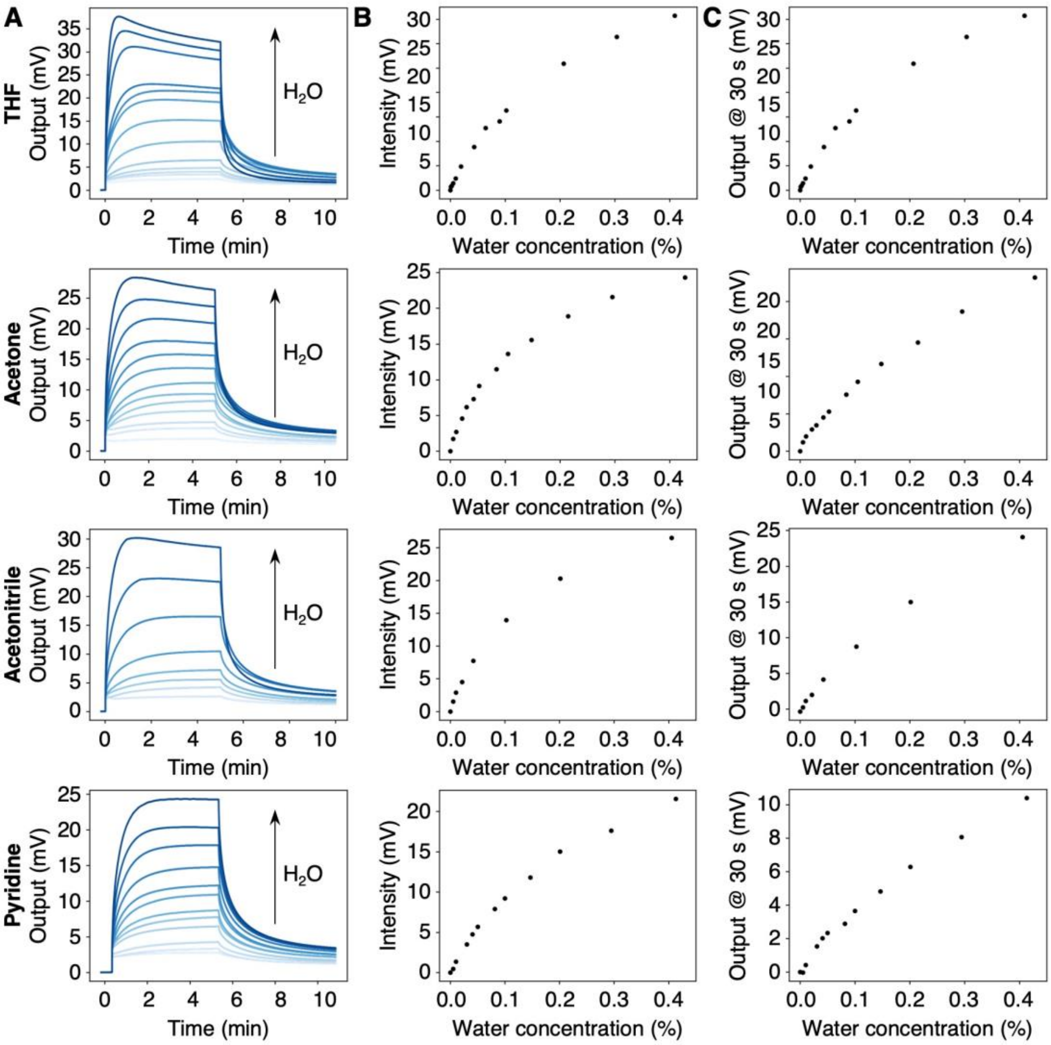

Figure S5. Signal responses to trace amount of water in various organic solvents.

A, Concentration-dependent signal responses to water in organic solvents: THF, acetone, acetonitrile, and pyridine. **B,C**, Plots of signal intensities (B) and output at 30 s after vapor injection (C) as a function of water concentration (w/w%).

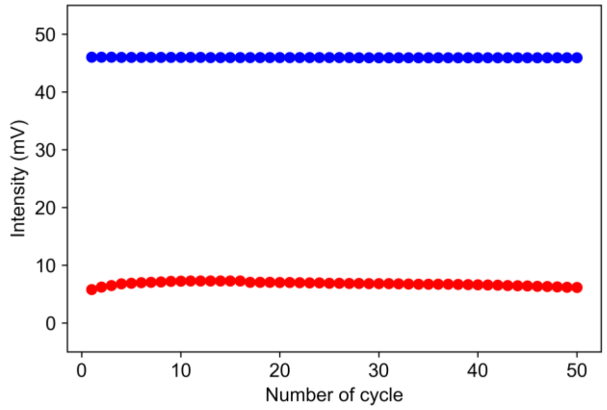

Figure S6. Reproducibility of DNA-based MSS.

Plots of signal intensities to water vapor (80%RH, blue) and vapor of water in THF (100 ppm, red).

Supplementary Table

Table S1. Relative sensitivity and Limit of Detection (LoD) of relative humidity sensors reported in literature.

Device	Receptor material	LoD [%RH]	Reference
Surface acoustic wave resonator (SAW)	MWCNT/nafion	0.5	[1]
Capacitive ultrasonic transducer (CMUT)	GO	0.4	[2]
Quartz crystal microbalance (QCM)	ZnO nanotetrapods	0.3	[3]
Surface acoustic wave resonator (SAW)	GO/PVA/SiO_2	0.25	[4]
Microcantilever, dynamic mode	ZnO-based cantilever	0.20	[5]
Film bulk acoustic wave resonator (FBAR)	ZnO	0.12	[6]
Membrane-type surface stress sensor (MSS)	CAB	0.09	[7]
Membrane-type surface stress sensor (MSS)	PEI	0.05	[8]
Quartz crystal microbalance (QCM)	TiO_2 nanoparticles	0.04	[9]
Microcantilever, dynamic mode	Hydrogel	0.03	[10]
Membrane-type surface stress sensor (MSS)	PEI	0.02	[8]
Microcantilever, dynamic mode	PMMA	0.02	[11]
Membrane-type surface stress sensor (MSS)	GO	0.019	[12]
Quartz crystal microbalance (QCM)	Cellulose nanocrystal	0.015	[13]
Membrane-type surface stress sensor (MSS)	PSS	0.008	[14]
Quartz crystal microbalance (QCM)	GO/PEI film	0.0069	[15]
Microcantilever, dynamic mode	CMOS metal	0.005	[16]
Capacitive micromachined ultrasonic transducer (CMUT)	Mesoporous silica	0.0014	[17]
**Membrane-type surface stress sensor (MSS)**		**0.0017**	**This work**

Abbreviations: CAB, cellulose acetate butyrate; GO, graphene oxide; MWCNT, multi-wall carbon nanotube; PEI, poly(ethylene imine); PSS, poly(sodium 4-styrenesulfate); PVA, poly(vinyl alcohol)

Table S2. Limit of Detection (LoD) of DNA-based MSS to trace amount of water in organic solvents.

**Solvent**	LoD	
	(w/w%)	(ppb)
THF	0.000053	530
Acetone	0.000082	820
Acetonitrile	0.000076	760
Pyridine	0.000120	1200

Supplementary References

1. Sheng, L.; Dajing, C.; Yuquan, C., A surface acoustic wave humidity sensor with high sensitivity based on electrospun MWCNT/Nafion nanofiber films. *Nanotechnology* **2011**, *22*, 265504. 10.1088/0957-4484/22/26/265504.
2. Sun, C.; Shi, Q.; Yazici, M.S.; Lee, C.; Liu, Y., Development of a Highly Sensitive Humidity Sensor Based on a Piezoelectric Micromachined Ultrasonic Transducer Array Functionalized with Graphene Oxide Thin Film. *Sensors* **2018,** *18*. 10.3390/s18124352.
3. Wang, X.-H.; Ding, Y.-F.; Zhang, J.; Zhu, Z.-Q.; You, S.-Z.; Chen, S.-Q.; Zhu, J., Humidity sensitive properties of ZnO nanotetrapods investigated by a quartz crystal microbalance. *Sens. Actuators B: Chem.* **2006**, *115*, 421–427. 10.1016/j.snb.2005.10.005.
4. Su, Y.; Li, C.; Li, M.; Li, H.; Xu, S.; Qian, L.; Yang, B., Surface acoustic wave humidity sensor based on three-dimensional architecture graphene/PVA/SiO2 and its application for respiration monitoring. *Sens. Actuators B: Chem.* **2020**, *308*, 127693. 10.1016/j.snb.2020.127693.
5. Mistry, K.; Nguyen, V.H.; Arabi, M.; Ibrahim, K.H.; Asgarimoghaddam, H.; Yavuz, M.; Munoz-Rojas, D.; Abdel-Rahman, E.; Musselman, K.P., Highly Sensitive Self-Actuated Zinc Oxide Resonant Microcantilever Humidity Sensor. *Nano Lett.* **2022**, *22*, 3196–3203. 10.1021/acs.nanolett.1c04378.
6. Qiu, X.; Tang, R.; Zhu, J.; Oiler, J.; Yu, C.; Wang, Z.; Yu, H., Experiment and theoretical analysis of relative humidity sensor based on film bulk acoustic-wave resonator. *Sens. Actuators B: Chem.* **2010**, *147*, 381–384. 10.1016/j.snb.2010.04.012.
7. Loizeau, F.; Akiyama, T.; Gautsch, S.; Vettiger, P.; Yoshikawa, G.; de Rooij, N., Membrane-Type Surface Stress Sensor with Piezoresistive Readout. *Proc. Eng.* **2012**, *47*, 1085–1088. 10.1016/j.proeng.2012.09.339.
8. Yoshikawa, G.; Akiyama, T.; Gautsch, S.; Vettiger, P.; Rohrer, H., Nanomechanical membrane-type surface stress sensor. *Nano Lett.* **2011**, *11*, 1044–1048. 10.1021/nl103901a.
9. Addabbo, T.; Fort, A.; Mugnaini, M.; Vignoli, V.; Baldi, A.; Bruzzi, M., Quartz-Crystal Microbalance Gas Sensors Based on TiO2Nanoparticles. *IEEE Transactions on Instrumentation and Measurement* **2018**, *67*, 722–730. 10.1109/tim.2017.2785118.

10. Xu, J.; Bertke, M.; Li, X.; Mu, H.; Zhou, H.; Yu, F.; Hamdana, G.; Schmidt, A.; Bremers, H.; Peiner, E., Fabrication of ZnO nanorods and Chitosan@ZnO nanorods on MEMS piezoresistive self-actuating silicon microcantilever for humidity sensing. *Sens. Actuators B: Chem.* **2018**, *273*, 276–287. 10.1016/j.snb.2018.06.017.
11. Karabacak, D.M.; Brongersma, S.H.; Crego-Calama, M., Enhanced sensitivity volatile detection with low power integrated micromechanical resonators. *Lab. Chip* **2010**, *10*, 1976–1982. 10.1039/b926170b.
12. Imamura, G.; Minami, K.; Shiba, K.; Mistry, K.; Musselman, K.; Yavuz, M.; Yoshikawa, G.; Saiki, K.; Obata, S., Graphene Oxide as a Sensing Material for Gas Detection Based on Nanomechanical Sensors in the Static Mode. *Chemosensors* **2020**, *8*, 82. 10.3390/chemosensors8030082.
13. Yao, Y.; Huang, X.-h.; Zhang, B.-y.; Zhang, Z.; Hou, D.; Zhou, Z.-k., Facile fabrication of high sensitivity cellulose nanocrystals based QCM humidity sensors with asymmetric electrode structure. *Sens. Actuators B: Chem.* **2020,** *302*. 10.1016/j.snb.2019.127192.
14. Yoshikawa, G.; Akiyama, T.; Loizeau, F.; Shiba, K.; Gautsch, S.; Nakayama, T.; Vettiger, P.; de Rooij, N.F.; Aono, M., Two dimensional array of piezoresistive nanomechanical Membrane-type Surface Stress Sensor (MSS) with improved sensitivity. *Sensors* **2012**, *12*, 15873–15887. 10.3390/s121115873.
15. Yuan, Z.; Tai, H.; Ye, Z.; Liu, C.; Xie, G.; Du, X.; Jiang, Y., Novel highly sensitive QCM humidity sensor with low hysteresis based on graphene oxide (GO)/poly(ethyleneimine) layered film. *Sens. Actuators B: Chem.* **2016**, *234*, 145–154. 10.1016/j.snb.2016.04.070.
16. Verd, J.; Sansa, M.; Uranga, A.; Perez-Murano, F.; Segura, J.; Barniol, N., Metal microelectromechanical oscillator exhibiting ultra-high water vapor resolution. *Lab. Chip* **2011**, *11*, 2670–2672. 10.1039/c1lc20103d.
17. Lee, H.J.; Park, K.K.; Kupnik, M.; Melosh, N.A.; Khuri-Yakub, B.T., Mesoporous thin-film on highly-sensitive resonant chemical sensor for relative humidity and CO2 detection. *Anal. Chem.* **2012**, *84*, 3063–3066. 10.1021/ac300225c.
